# Ultra-High-Throughput Absorbance-Activated Droplet Sorting for Enzyme Screening at Kilohertz Frequencies

**DOI:** 10.1021/acs.analchem.2c04144

**Published:** 2023-02-27

**Authors:** Elliot J. Medcalf, Maximilian Gantz, Tomasz S. Kaminski, Florian Hollfelder

**Affiliations:** † Department of Biochemistry, University of Cambridge, 80 Tennis Court Road, CB2 1GA Cambridge, United Kingdom; ‡ Department of Molecular Biology, Institute of Biochemistry, Faculty of Biology, University of Warsaw, Miecznikowa 1, 02-096 Warsaw, Poland

## Abstract

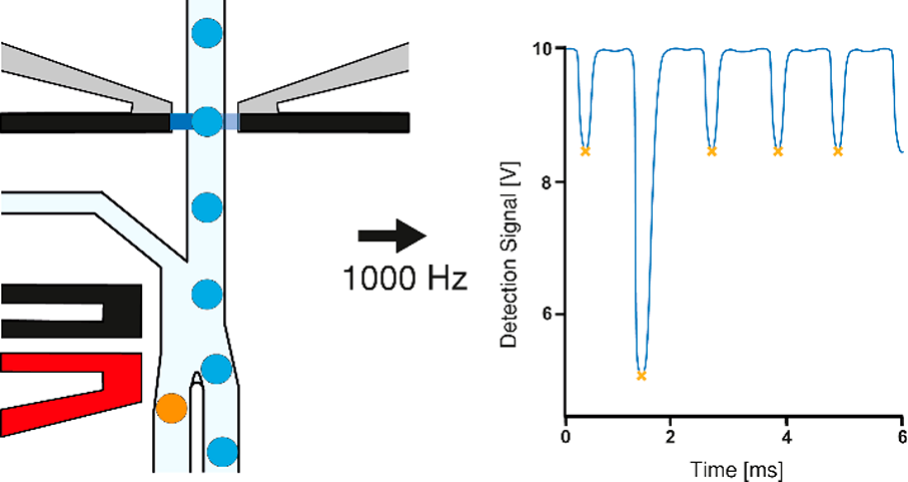

Droplet microfluidics is a valuable method to “beat the odds” in high throughput screening campaigns such as directed evolution, where valuable hits are infrequent and large library sizes are required. Absorbance-based sorting expands the range of enzyme families that can be subjected to droplet screening by expanding possible assays beyond fluorescence detection. However, absorbance-activated droplet sorting (AADS) is currently ∼10-fold slower than typical fluorescence-activated droplet sorting (FADS), meaning that, in comparison, a larger portion of sequence space is inaccessible due to throughput constraints. Here we improve AADS to reach kHz sorting speeds in an order of magnitude increase over previous designs, with close-to-ideal sorting accuracy. This is achieved by a combination of (i) the use of refractive index matching oil that improves signal quality by removal of side scattering (increasing the sensitivity of absorbance measurements); (ii) a sorting algorithm capable of sorting at this increased frequency with an Arduino Due; and (iii) a chip design that transmits product detection better into sorting decisions without false positives, namely a single-layered inlet to space droplets further apart and injections of “bias oil” providing a fluidic barrier preventing droplets from entering the incorrect sorting channel. The updated ultra-high-throughput absorbance-activated droplet sorter increases the effective sensitivity of absorbance measurements through better signal quality at a speed that matches the more established fluorescence-activated sorting devices.

## Introduction

Functional screening of millions of different proteins in one experiment is possible at ultrahigh throughput in droplet microfluidics, where water-in-oil emulsions cocompartmentalize genotype and phenotype. A selection is achieved by interrogating these pico- to nanoliter-sized “test tubes” for an assay reaction that brings about an optically active primary or downstream product. Several successfully directed evolution campaigns have been performed using droplet microfluidics to improve enzyme variants and develop non-natural catalytic properties.^1−5^ Additionally, droplet microfluidics has also been used for functional metagenomics,^6−8^ strain enrichment,^9^ epistatic mapping,^10^ and single-cell analysis.^11^ Fluorescence assays are the most sensitive format for product detection: in fluorescence-activated droplet sorting (FADS), a few thousand molecules of a fluorophore in a droplet (corresponding to the nM range, e.g., fluorescein) can be detected with kHz rates.^12,13^ FADS is the most popular method for performing directed evolution campaigns using droplet microfluidics.^13^ However, the fluorogenic assays only cover a fraction of the reactions of interest, so alternative detection modes are required. Fluorescence-based assays are not available for many enzymatic assays, and they require synthetic substrates that are typically large and hydrophobic to accommodate the fluorophore. In a directed evolution campaign, these features can favor binding to the artificial fluorophore rather than the core substrate. Absorbance-activated cell sorting (AADS) provides practical means to cover chromogenic assays,^14−18^ offering the opportunity to test a more comprehensive array of substrates.

Practically AADS is attractive: the setup is more straightforward and less expensive than FADS, as no lasers and photomultiplier tubes are needed. These factors also reduce safety requirements and mean that the microfluidic rig can be run on a lab bench instead of in a dedicated laser room. On the other hand, detection for AADS is not as sensitive as fluorescence detection (high μM vs nM detection limits, respectively).^12^ The AADS droplet sorter developed by Gielen et al. has been applied to the directed evolution of phenylalanine dehydrogenase^14,19^ and glucose dehydrogenase.^20^ Additional improvements to this initial design have been made: Zurek et al. overcame sensitivity limitations in droplet absorbance measurements by the growth of clonal variants in droplets.^17^ Duncombe et al. introduced UVADS (UV–vis spectra-activated droplet sorter), which allows the collection of the whole spectra from 200 to 1050 nm, including a right-angled turn at the detection interface to increase the droplet path length for higher sensitivity.^15^ However, the main limitation in all AADS designs remains the throughput at which droplets can be reliably sorted. The highest claimed sorting rate for absorbance detection is 300 Hz^14^ (although most enzymatic screening campaigns in our experience are generally performed at a lower throughput, e.g., 100 Hz, due to a higher likelihood of incorrect droplet sorting at higher frequencies). This trade-off poses a limitation on the size of a library that can be screened in a practical time frame and constrains the scope of the absorbance detection technology when trying to obtain rare variants in a screening campaign.

Compared to FADS, the second disadvantage of current AADS setups is that absorbance is directly proportional to path length, meaning that droplets with larger diameters and, therefore, larger volumes are needed. This, in turn, increases the amount of reagent required for each droplet and limits the throughput of sorting since a higher electric field is needed to sort larger droplets. If the electric field is too large, droplets tend to merge and/or become fragmented. A remedy to prevent merging is to provide a “Faraday moat”^21^ surrounding the channels upstream and downstream of the sorting junction; however, fragmentation remains an issue. A third problem is of a practical nature, namely that the scattering caused by droplet edges as they pass the optic fibers places limits on the minimum droplet size and minimum substrate concentration that can be quantitatively detected. This effect becomes more pronounced for lower concentrations of the absorbing medium, as scattering obscures the true value of absorbance for lower molarities (i.e., the absorbance value is “hidden” in between the edges). A key challenge in directed evolution is the notion of “beating-the-odds”, such that the more variants screened, the higher are the chances of improving functional performance. Currently, FADS is at least ∼20-fold faster than AADS (1–2^1,3,6,7^ kHz vs 100 Hz^14^), implying a substantially reduced number of potential variants screened and leaving much sequence space unexplored.

In the present work, we systematically explore various microfluidic, chemical and computational features of AADS and introduce improvements that collectively level its throughout with FADS, while still retaining high sensitivity, albeit at a higher volume than with a typical FADS setup. Specifically, we address (i) improved microfluidic design, (ii) the use of added compounds for refractive index matching that are mixed with the spacing oil, and (iii) development of new software for signal detection and automation of droplet sorting. Finally, the utility of our improved UHT-AADS (ultra-high-throughput AADS) was validated in an enrichment of phenylalanine dehydrogenase (PheDH) for the conversion of l-phenylalanine (l-Phe) to phenylpyruvate.

## Materials and Methods

### Chip Fabrication

Microfluidic chips were fabricated using soft lithography in two layers. Briefly, microchannels were obtained from polydimethysiloxane (PDMS) and were bonded to glass slides after surface plasma treatment. Device designs are deposited on our repository DropBase.^22^ The detailed fabrication protocol is provided in the SI.

### Droplet Generation and Sorting

Droplets were made with a flow-focusing microfluidic junction using HFE-7500 (3 M Novec; refractive index *n*_D_ = 1.29)^23^ fluorinated oil and 2% 008-FluoroSurfactant (RAN Biotechnologies). Gas-tight syringes (Hamilton Company) were operated with syringe pumps (Nemesys, Cetoni). Droplets were sorted into the positive outlet channel by dielectrophoresis. Electric pulses were applied using on-chip NaCl electrodes. Optical fibers were used to pass light from a LED (455 nm, M455F3, Thorlabs) via the detection point to a photodetector (PDA36A, Thorlabs). Data visualization and triggering of sorting events were performed as previously described by Gielen et al.^14^ A detailed protocol is provided in the SI. 1,3-Bis(trifluoromethyl)-5-bromobenzene (Sigma; refractive index *n*_D_ = 1.427) was mixed with HFE-7500 at different volume percentages, as described in the Results and Discussion section. Flow rates and sorting parameters are provided in the SI.

### Droplet Sorting Algorithm

The code is modified from Zurek et al.^1^ and is written in the C programming language and was written to maximize the speed of computation and run on an Arduino Due. Constant integer types were used, blocks of code were wrapped in functions, and the “micros” function was used to allow continuous running of code with delays. A detailed description of alterations and the full code can be found in the SI and on GitHub.^24^

### Enrichment of a Variant Expressing Phenylalanine Dehydrogenase

The enrichment experiment was carried out as previously described by Gielen et al.^14^ and a detailed protocol is provided in the SI. Briefly, a wild-type PheDH construct as positive control and a glycosidase as a negative control was expressed in *E. coli*. A 1:100 dilution of positive to negative control was compartmentalized with substrate solution in a flow-focusing droplet generation device. Droplets were incubated overnight and the positive fraction was sorted at 1 kHz (see SI, section S2 for parameters). After sorting, the emulsion was broken and plasmid DNA was extracted and transformed into *E. coli*. Single colonies were picked and grown to saturation in 96-well plates. After protein expression, cells were lysed. Enzyme assays with 10 mM l-phenylalanine and 10 mM NAD (Sigma) were conducted in 96-well microplates. Absorption at 340 nm was measured after incubation for 20 h. For calculating enrichment factors, equations previously used by Baret et al.^25^ and Zinchenko et al.^26^ were employed.

## Results and Discussion

### Device Design for Increased Droplet Throughput

**Figure 1:**
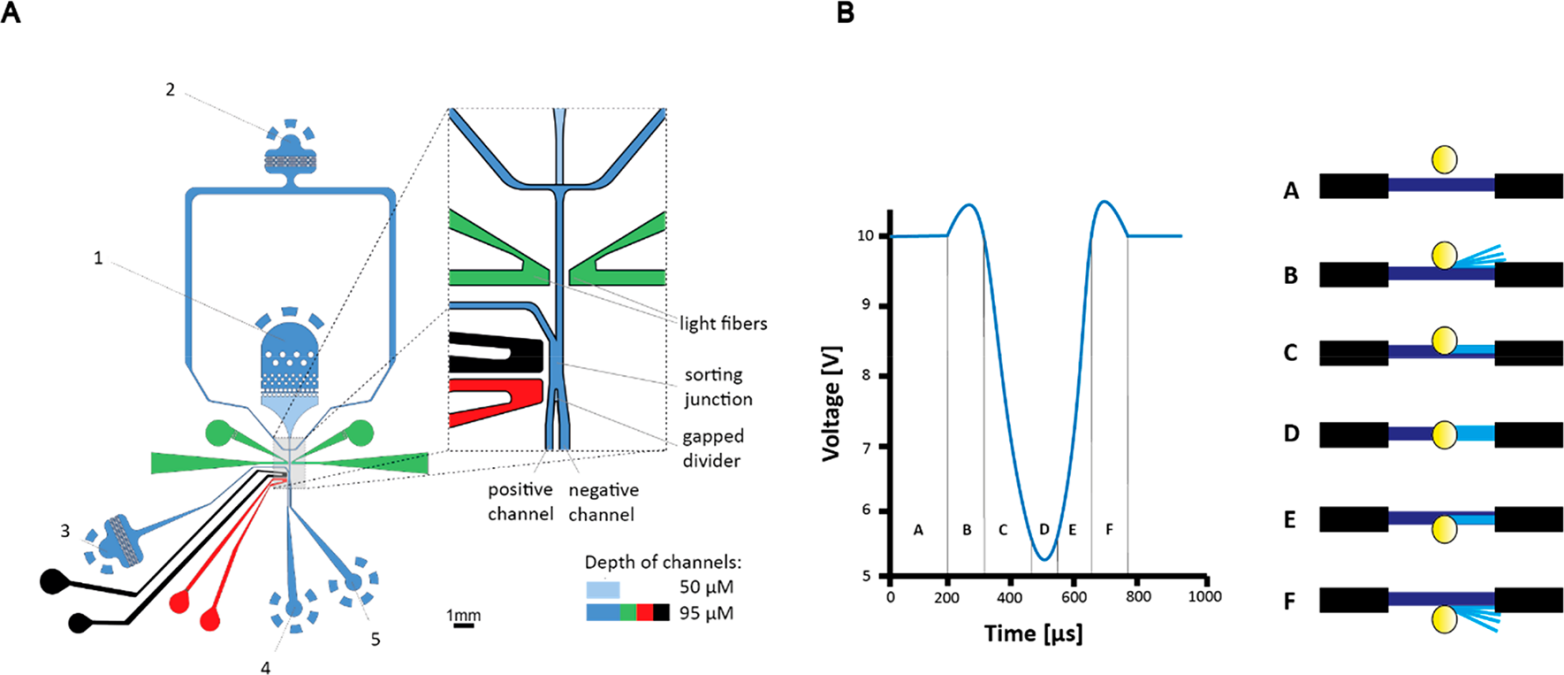
(A) Schematic of the UHT-AADS showing the single 50 μm layer channel (light blue) and a close-up design of the sorting junction showing the gapped divider at the bifurcation (light blue). The gapped divider is on the second 50 μm layer: (1) input channel for droplets, (2) input channel for spacing oil, (3) input channel for bias oil, (4) positive channel outlet, (5) negative channel outlet, (6). The ground electrode is colored black and the positive electrode is colored red. Adding a single-layer droplet injection chamber allows for even spacing of the 75 pL droplets. The bias oil channel acts as a barrier to droplets from entering the negative chamber unless acted on by the dielectrophoretic force. (B) A diagram showing a typical droplet trace as it passes the optical fibers. At position A, light transmission is at the maximum value indicated by the baseline value set. At B, the droplet edge causes refraction at the water–oil interface producing a “shoulder” corresponding to the higher amount of light collected by the fiber. At C, the droplet is moving toward the center of the optical fibers. At D, the droplet is in the center of the optic fiber, and the true peak value of absorbance (minimum amount of light) is given in the droplet trace. At E, voltage increases as the droplet moves away from the optical fiber center. At F, there is the effect of the other droplet edge causing another peak (or a “shoulder”) of collected light.

To achieve higher sorting frequencies, we first optimized the microfluidic sorting device through several iterations of design, fabrication, and testing of its performance. The design of a final device suitable for higher throughput is shown in Figure 1A. The two-layer chip features a bias oil inlet (see 3 in Figure 1A) for better spacing of reinjected droplets and also a gapped divider (see close-up in Figure 1) at the sorting junction to minimize droplet fragmentation (adapted from Sciambi et al.^21^). The partial barrier (gapped divider) between the two outlets is designed so that the droplets do not break apart on impact and instead “hug” the sides of the barrier. This is important for sorting of larger-sized droplets at higher speeds since a gentler impact ensures droplet stability. Large volumes inherently limit the sorting speed since as the volume increases, the electrophoretic forces required to move the droplet increase,^14^ so smaller droplet volumes were employed in our study. Since absorbance is directly proportional to path length, in line with the Beer–Lambert law, decreasing droplet volumes reduces the diameter of the droplets and therefore leads to a lower absorbance that is harder to detect. There is, therefore, a trade-off between the droplet size and the maximum sorting speed. Also, as the droplet size increases, due to the shear forces acting on them, droplets are more likely to fragment due to the higher flow rate and the higher electric field needed to direct droplets to the positive channel.

The depth of the AADS device is determined by the width of the optical fibers. By using a shallower 50 μm deep droplet injection chamber, smaller droplets can be evenly spaced, preventing two droplets from being injected at the same time and sorted incorrectly. These smaller droplets in a device with a depth of 100 μm are not squeezed, and therefore, “derailing” them in the sorting junction becomes easier so that higher sorting frequencies are achieved, which is also demonstrated in this study. The effect of a decreased droplet size and increased sorting speed is evaluated in the following experiments. Higher throughput of optimized microfluidic devices required implementation of improvements in the software that was used for recording the signal and triggering sorting events (detailed in the SI).

### Refractive Index Matching of the Oil Phase Creates Smoother Voltage Peak Shapes

**Figure 2:**
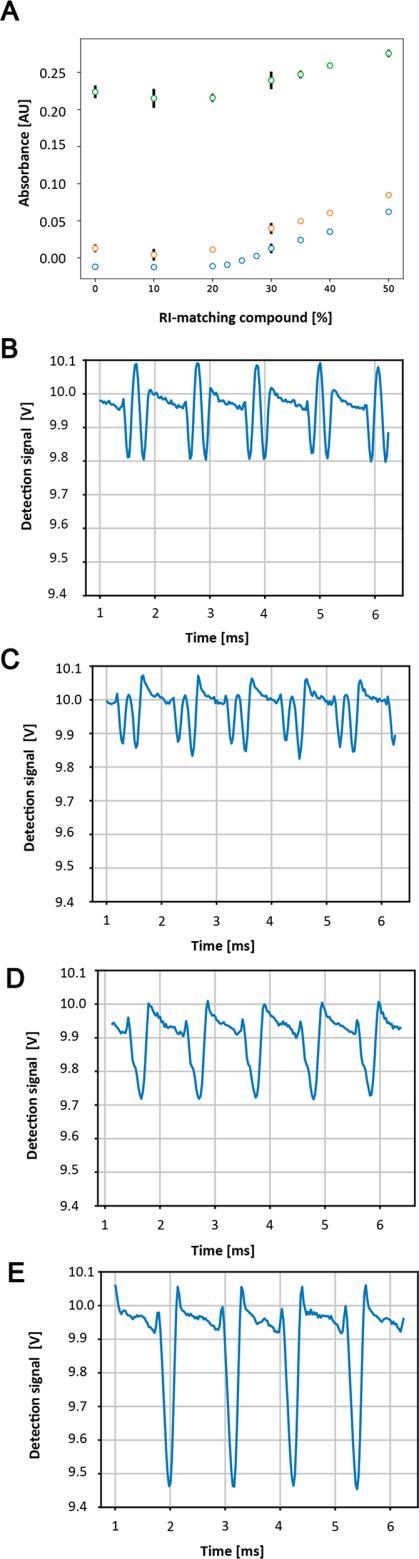
(A) Effect of absorbance (455 nm) against different percentages of RI-matching oil for different concentrations of the absorbing compound. Blue points are 0 mM tartrazine, orange are 0.5 mM tartrazine, and green are 5 mM tartrazine. Different tartrazine concentrations were used to assess the dynamic range. As the RI-matching oil percentage increases, the absorbance also increases. The RI-matching oil percentage is the percentage volume of the additive added to the HFE-7500 oil. Each point corresponds to data from 20 s of droplet voltage recordings at 1000 Hz. Error bars are the standard deviation of the 20000 droplet peaks extracted from the data. (B–E) 6 ms droplet detection signal [V] for 0 mM tartrazine (water) droplets using (B) 25% RI-matching oil, (C) 27.5% RI-matching oil, (D) 30% RI-matching oil, and (E) 35% RI-matching oil. Droplet peaks are clearly distinguished from the baseline, and droplet edges have been removed, with a clear true peak at the center of each droplet trace. See section S4 (SI) for analogous droplet traces converted into absorbance values.

**Figure 3:**
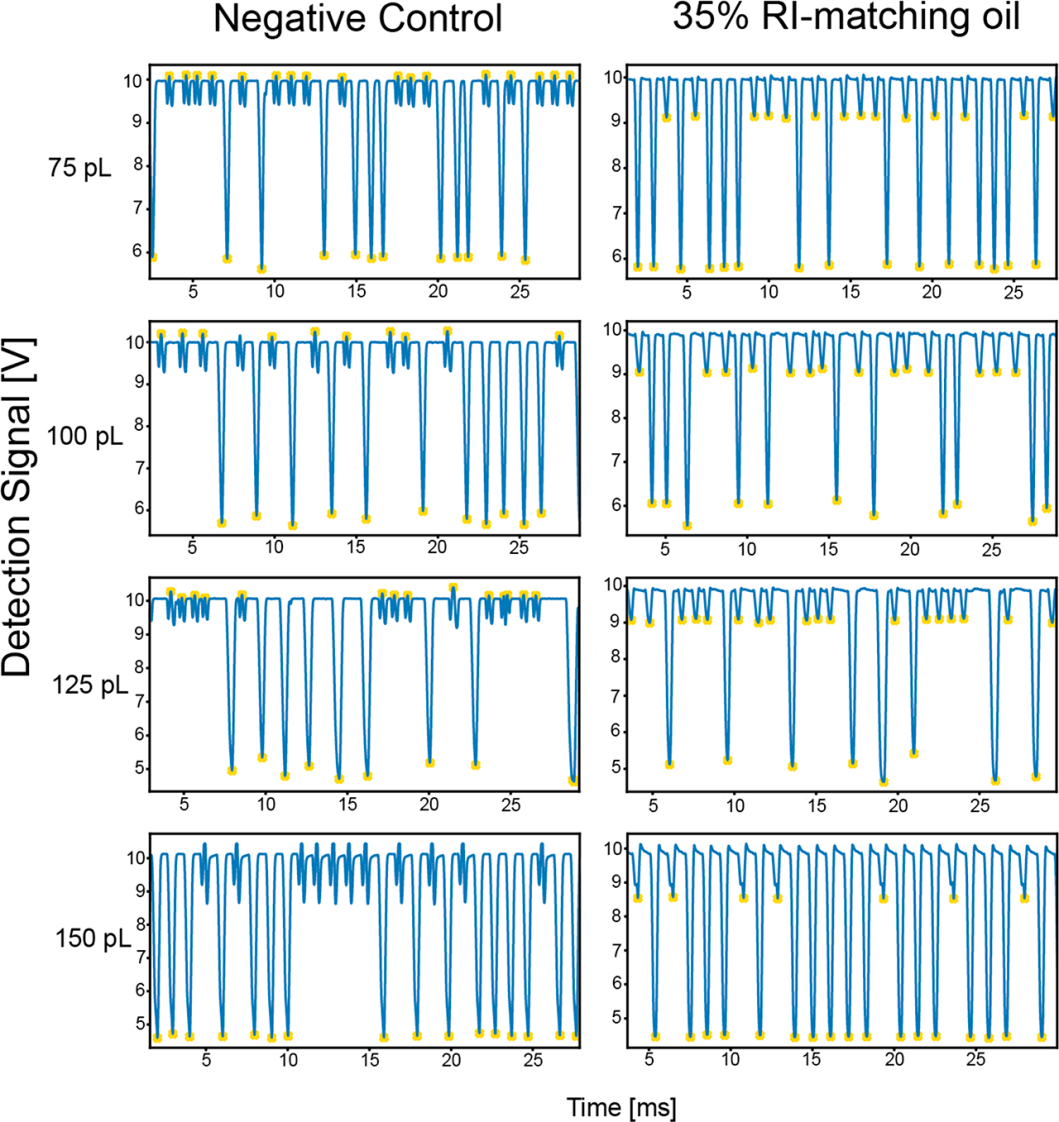
Effect of RI-matching oil on the droplet traces (V) at different droplet sizes with two droplet populations, 0.5 mM tartrazine (peaks at higher voltage) and 5 mM tartrazine (peaks at lower voltage) at approximately 1 kHz frequency of droplet measurement. The negative control is without 1,3-bis(trifluoromethyl)-5-bromobenzene and shows significant droplet edges for all droplet volumes. No droplet edges are seen when using 35% RI-matching oil, and peaks are clearly distinguishable. Droplets with a volume of 75 pL are distinguishable at 0.5 mM. The yellow asterisks show droplet peaks identified using a custom peak detection algorithm (SI). The algorithm cannot distinguish the droplet values for the 150 pL negative control trace due to broad peaks leading to an unidentifiable maximum.

We additionally focused on implementing a new strategy for the removal of unwanted scattering signals coming from light deflected by the droplet interfaces. The determination of the true absorbance value becomes much more challenging due to the presence of the spikes caused by the droplet edges. This makes it algorithmically harder to assign a true absorbance value correctly (see Figure 3, 150 pL negative control) and takes additional time to compute the correct value, complicating real-time signal processing. Specifically, readouts of the droplet absorbance by monitoring voltage over time showed scattering at the droplets’ edges at the oil–water interface of droplets as they pass through the optical fiber detection area (Figure 2B,C). This effect is due to refraction and causes unwanted spikes in the voltage data, so that the true absorbance value of the droplet is only visible in between the spikes. So far, the problem of spikes was bypassed by the addition of a dye offsetting the signal (Gielen et al.^14^). At the saturation limit of the photodetector, droplets with low molarity of absorbing dye can have an absorbance value greater than that of the saturation limit, lifting the true value above the saturation limit (rendering it nonquantitative). To minimize this effect, an offset of tartrazine can be added to the droplet contents, resulting in a deliberate absorbance increase to a level below the saturation limit. However, doing so reduced the dynamic range, as the upper limit of the detection range was reached earlier in the presence of added tartrazine.

In an alternative approach to mitigating the issue of scattering at droplet edges, 1,3-bis(trifluoromethyl)-5-bromobenzene was added to the fluorocarbon HFE-7500 oil (Salmon et al.^27^) as an example of a refractive index (RI) matching compound. This RI-matching oil has high miscibility with oil and does not interfere with droplet reinjection and spacing in the sorting junction. Several additional practical considerations determined how such a mixture best removed the droplet edges in the signal, provided a wide dynamic range, and allowed empty droplets to be confidently identified. The RI-matching oil had an additional effect on the measured absorbance values. The percentage is the percentage volume of 1,3-bis(trifluoromethyl)-5-bromobenzene added to HFE-7500 oil. The choice of a suitable percentage of RI-matching agent is determined first by trying to minimize the scattering effect, and second to have a suitable dynamic range between high and low concentrations of absorbing compound (e.g., tartrazine). Figure 2A shows how increasing the percentage of 1,3-bis(trifluoromethyl)-5-bromobenzene leads to an increase in voltage, although the absorbance of tartrazine, here used as a model absorbing compound, does not change. Indeed, the gradient is increased for 0.5 and 0 mM tartrazine concentrations beyond 30% RI-matching oil. This trend indicates that increasing the concentration of RI-matching oil decreases the assay’s dynamic range since there is a lower range between, e.g., 5 and 0.5 mM tartrazine at higher concentrations of RI-matching oil. Figure 2B,C shows that for a RI-matching oil concentration of 25% and 27.5% there are still significant droplet edges, and the true peak lies in between. Values with a RI-matching oil percentage (percentage volume of the additive added to the oil) of greater than 30% (Figure 2D,E) do not show droplet edges in the trace, and therefore, their true value is easy to deconvolute. However, for a RI-matching oil concentration of 30% there are asymmetries in the droplet trace, potentially leading to uncertainty over the true value. It is necessary to detect empty droplets for frequency and other measurements (e.g., gating in directed evolution screening campaigns), so a suitable concentration of RI-matching oil should also give enough of a voltage signal to allow a signal to be detectable for these droplets (albeit without droplet edges).

Overall, the value chosen was 35% 1,3-bis(trifluoromethyl)-5-bromobenzene since this represents the best compromise between removing droplet edges, providing a high dynamic range, and allowing empty droplets to be identified. Removing edges significantly minimizes the sorting algorithm’s complexity (since it is difficult to deconvolute the true value from traces with edges) and therefore increases computation speed.

The addition of a RI-matching compound to the spacing oil also required a change in the design of the microfluidic sorting junction. Since the RI-matching oil reduces emulsion stability and might cause the wetting of droplets to walls of channels or tubing, we decided to apply a strategy of transferring positively sorted droplets back to the oil without RI. Therefore, a bias oil channel (through which standard HFE-7500 oil with 0.5% 008-FluoroSurfactant, i.e., with no RI-matching compound) was added at the sorting junction and during sorting, both oils are in a laminar flow regime. This standard oil, therefore, poses a barrier to droplets from entering the positive outlet. Only when the electric pulse applied to the droplets from the sorting electrode exceeds the inertial force driving the droplet into the negative outlet does the droplet enter the positive outlet (see Figure 1A for the diagram).

### Effect of Droplet Size on Absorbance

Figure 3 compares the effect of different volumes for two droplet populations (0.5 and 5 mM tartrazine) measured at 1 kHz with and without 35% RI-matching added to the spacing oil. As the droplet volume decreases for both the negative control and 35% RI-matching oil, the detected voltage also decreases, potentially due to the decreased path length of the droplet resulting in a lower absorbance value. As shown in Figure 3, the droplet traces for 75 pL droplets with RI-matching oil show clear peaks with no shoulders. However, without RI-matching oil, the scattering effect causes the true droplet value to be above the baseline and is masked by the higher absorbance of the spacing oil. The measurement, therefore, is not quantitative as the droplet values are past the saturation limit.

As previously stated, droplets with a smaller volume are easier to sort at high frequencies due to less force needed to move them effectively. Our results proved that modified microfluidic design and adding RI-matching oil allow for detection and sorting of droplets with a volume of 75 pL. This is a significant improvement compared to larger volumes of droplets in previous studies (e.g., 180 pL in Gielen et al.^14^)

### Validation of Sorting Efficiency at Different Droplet Frequencies

To test whether the improvements described in the previous paragraphs allow not only fast sorting, but also high efficiency in the sorting outcome, we carried out selections (between two 75 pL droplet populations, 0.5 and 5 mM of tartrazine, respectively) at different frequencies (1, 1.5, and 2 kHz). Flow rates were adjusted to allow different droplet frequencies, and the ratio of the flow rates was kept the same between frequencies (1:5:10 ratio for droplet/bias oil/spacing oil, respectively). Droplets were counted as being correctly sorted only if the droplet triggering the sorting event was sorted alone, without any fragments from other droplets before or following behind. Droplets are in the RI-matching oil only for a very brief period between respacing in the channel just upstream of the sorting junction and exiting to the positive sorting channel (where they mix with pure oil), and so the contact time of droplets with the RI-matching oil is miniscule. Furthermore, droplets are demulsified post-sorting.

**Figure 4:**
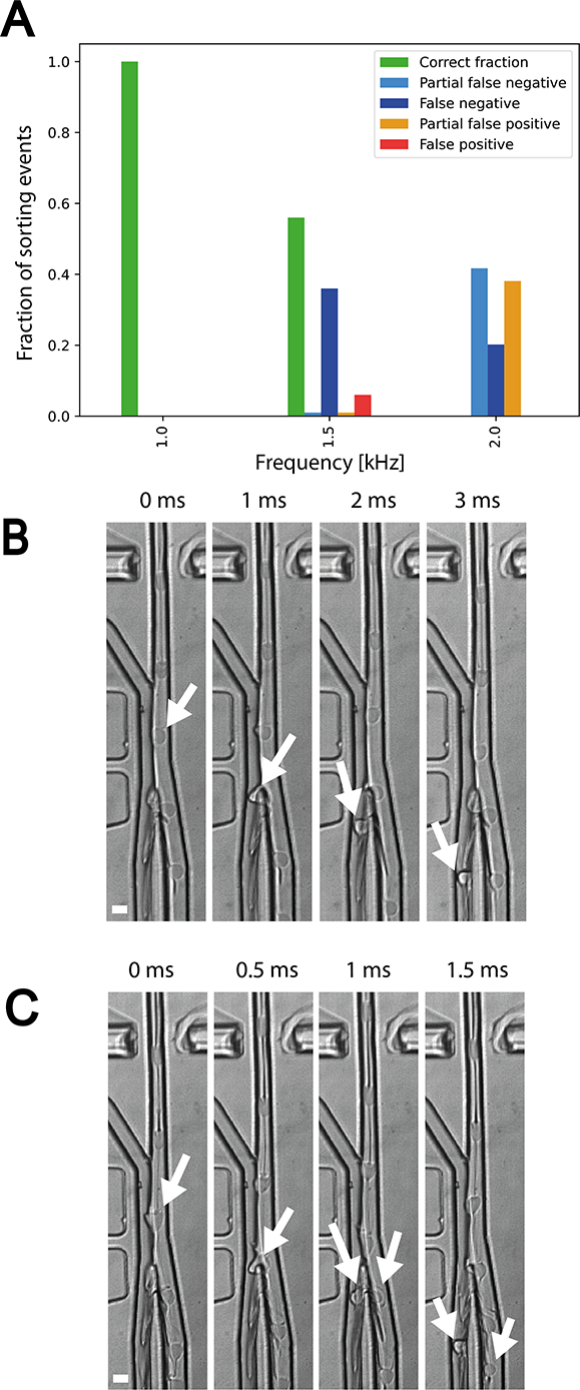
(A) Histogram showing the fraction of correctly sorted droplets, partial false negatives, false negatives, partial false positives, and false positives at 1, 1.5, and 2 kHz. Partial false negatives are droplets that are false negatives that split at the junction. Partial false positives are false positives that split at the junction. A high-speed camera was triggered at every sorting event, and visual inspection in ImageJ was used to determine if the droplet moved into the correct outlet. Droplets were counted as correctly sorted only if the droplet that triggered the event was sorted alone. The events examined were ∼100. (B) Snapshots of droplets at 1000 Hz when the sorting electrode is triggered based upon the correct absorbance value. The droplet that is correctly sorted is shown with the white arrows. A video of this experiment is available as Supporting Information. (C) An example of a droplet fragmenting at 2000 Hz due to collision with the central barrier. Arrows show the droplet splitting into two sub-volumes. Scale bars are 50 μM.

Fragmentation of droplets occurs as the frequency increases due to the insufficient force required to move the droplet into the positive outlet, and the droplet collides with and breaks at the barrier. Additionally, as the electric field increases, this also leads to droplet fragmentation. This is also more pronounced because of the different oil composition at the sorting junction. Figure 4C shows an example of a sorting event recorded at a high frequency of 2 kHz, suggesting that the force required to move the droplet into the positive outlet chamber is not sufficiently strong: the droplet collides with the center of the barrier, splitting it into two daughter droplets. A potential remedy would be to use a greater electric field to move the droplet into the positive outlet more strongly before it collides with the barrier. However, this may also inadvertently cause the droplet to break up due to increased instability at the droplet surface.^28^

Finally, since the bias oil (coming in from the side channel) and flow rate of the droplets from the main channel are entering the junction at different rates, this poses an additional barrier to pulling the droplet into the positive channel. As the droplet is pulled over to the positive elution channel, part of it is still being pulled into the negative outlet at a higher flow rate. This stress on the droplet causes the droplet to break apart at the barrier.

When probing higher droplet frequencies, the forces needed to pull the droplet into the positive outlet chamber must become larger. Accordingly, sorting efficiency decreases as the droplet frequency increases (Figure 4A), and the number of droplets that become fragmented also increases (partial false negatives and false positives). All in all, a compromise between the high voltage needed to pull the droplet into position and a low enough voltage that does not cause droplet fragmentation is needed to achieve a balance between droplet and bias oil flow rate to ensure minimization of false positives but with still enough force to sort positive droplets correctly.

In the light of these issues, the success of sorting was evaluated by analyzing video traces (with ∼100 droplets after sorting) with an algorithm detecting the absorbance in droplets passing the detector. Sorting was carried out at 1 kHz (100% efficiency for 100 videos analyzed), and a 10-fold improvement of the apparatus used by Gielen et al.^14^ is seen. A video showing sorting at 1 kHz is provided in the Supporting Information.

### Validation of the Sorting Accuracy by Enrichment of Active Phenylalanine Dehydrogenase Variants in a Single Cell Lysate Screening Experiment at 1 kHz

**Figure 5:**
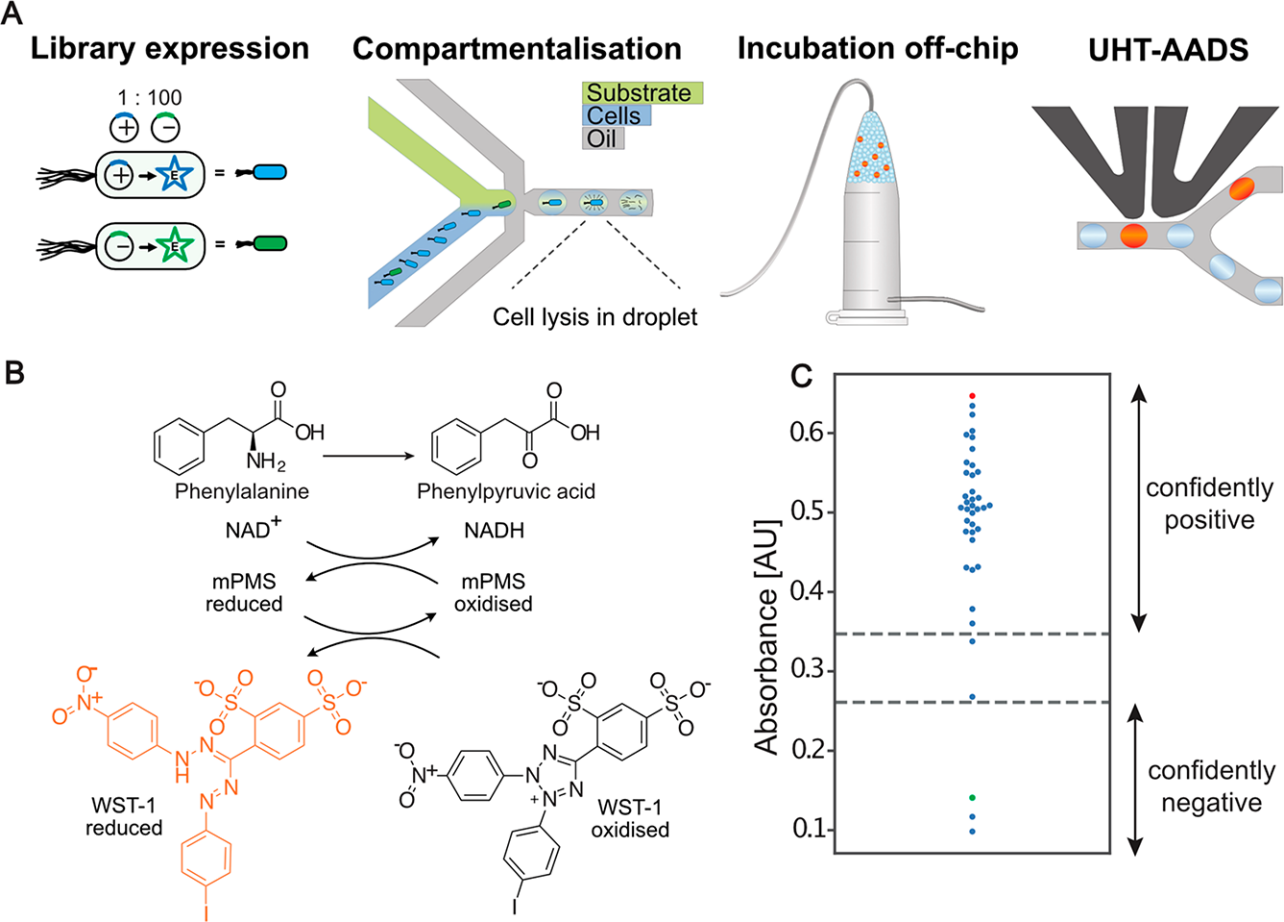
(A) Illustration of a screening workflow, showing *E. coli* expression of a library, droplet generation for single cell encapsulation of library members, incubation off-chip and sorting for active phenylalanine dehydrogenase (PheDH) activity using the novel UHTS-AADS device. (B) The coupled reaction produces reduced WST-1 with absorbance at 455 nm. (C) Swarm plot of the mean absorbance at 340 nm for 38 colonies (blue, *n* = 3 for each) picked after transformation of DNA collected from the positive outlet of the AADS device sorting at 1 kHz. The red dot shows the mean (*n* = 9) of the positive control (pASK expressing wild-type PheDH), and the green dot shows the mean (*n* = 9) of the negative control (pASK expressing glycosidase). The confidently positive clones are classified as two standard deviations from the mean of the positive control and respectively for the negative control (dotted lines).

Enrichment experiments are recognized as standard proof-of-principles experiments to assess the suitability of a device for enzyme discovery.^25,26^ To evaluate if UHT-AADS can be used to screen for phenylalanine dehydrogenase (PheDH) activity, we used a previously established droplet-based colorimetric cell lysate assay workflow^14^ and combined it with our novel device. We generated a mock library consisting of PheDH and a glycosidase as negative control mixed in a 1:100 ratio (Figure 5A,B). The mock library was encapsulated in monodisperse droplets with the substrates and lysis agent and incubated off-chip. We then screened for droplets positive for PheDH activity at the improved sorting rate of 1 kHz for 200 000 droplets, in this improved ultra-high-throughput assay (taking the definition of ultrahigh-throughput as being greater than 100 000 samples screened per day^29^). DNA was recovered by transformation into *E. coli* and a small sample was analyzed in a secondary photometric assay to evaluate the efficiency of sorting. A total of 34 of 38 clones tested showed PheDH activity (Figure 5C; within two standard deviations from the mean of the positive), giving a true positive rate of 89%. This translates into a 94-fold enrichment calculated according to Zinchenko et al.^26^ and a 1683-fold improvement according to Baret et al.,^25^ suggesting that the enrichments are suitable for functional selections.

## Conclusion

Absorbance-activated droplet sorting is still less frequently used for protein engineering^14,17^ compared to the more established FADS, despite their complementarity and the possibility of screening a larger and different pool of potential reactions. The introduction of RI-matching oil, a faster sorting algorithm, a single-layered inlet, and bias oil at the sorting junction enable AADS to catch up in its performance; the 1 kHz sorting rate (with 100% efficiency for 100 videos analyzed) is equivalent to FADS campaigns by employing the improvements discussed in this paper (see S14 for a table comparing the improvements to the previous designs). Based on this 10-fold improvement in throughput (compared to 100 Hz reported in biological experiments previously^14^) larger libraries can be screened in the same amount of time, increasing the chances of success by making it more likely to identify rare functional variants as larger fractions of sequence space are interrogated.

Other drawbacks of AADS remain: for FADS, the minimum product concentration is >2 nM (corresponding to >2500 product molecules per droplet), whereas for AADS the sensitivity is lower at >7.5 μM (with >10^9^ molecules per droplet).^13^ In fact, the sensitivity of the setup described in this work is slightly lower than that introduced by Gielen et al.^14^ due to the path length reduction (see S9 for a calibration curve). However, the local enzyme and product concentrations are larger in smaller droplets (2.4-fold in 75 vs 180 pL droplets), partially compensating for the reduced sensitivity. In-droplet growth of enzyme-expressing *E. coli* cells (after single cell encapsulation) has been shown to increase the amount of enzyme in an assay, which provides means to boost the product signal (while also reducing phenotypic variation).^17^ Increasing the sensitivity of the AADS device may be possible by choosing a readout molecule with a high extinction coefficient (e.g., gold nanoparticles^30^) For example, Probst et al.^16^ used nanoparticles, a combination of confocal optical systems and a postprocessing algorithm to achieve a sensitivity of 800 nM. As a suggestion for future improvements, broadband-enhanced cavity absorption with mirrors could be added,^31,32^ although manufacturing is complex due to needing to precisely place micromirrors on either side of the channel between the optical fibers.

On the other hand, AADS is cheaper to build (requiring no lenses and expensive laser equipment) than FADS, can be used on a benchtop (without laser protection) and provides a very accessible setup for ultrahigh throughput screening.^13^ The disadvantages of using RI-matching oil are potential instability and wetting problems, as previously mentioned. However, the droplets are only transferred to the RI-matching oil for a very brief period while sorting. The effect of being able to increase the sorting speed through smaller droplets using the RI-matching oil is a sufficient trade-off for a slightly decreased dynamic range and sensitivity. An alternative to RI-matching oil is to add a “baseline offset” in which a small amount of absorbing compound (e.g., tartrazine) is added to the aqueous phase. In this case, the signal from all the droplets is artificially increased above the baseline and therefore scattering effects are also minimized. The robustness of absorbance sorting is enhanced by these practical measures so that its use will make it easier to obtain quantitative data or good quality, and an increased sorting frequency ensures accurate sorting decisions based on high quality peak detection at lower volumes and high frequency. Postprocessing analysis of droplet data can additionally be easily reviewed through the Python scripts provided, and has been written with packages that are consistently improved by the community for easy updating of the code. Also, since the sorting uses Arduino-like microcontrollers, improvement in hardware will result in increased computation speed while retaining the accessibility of being inexpensive and open-source. Complementary work by Richter et al.^33^ has shown an increased sorting throughput and removal of droplet trace artifacts by using a combination of surface acoustic waves and microlenses in the form of an optical air cavity. We envision that a further upgrade to AADS designs could incorporate design improvements from both studies.

By making device designs (deposited on our repository DropBase as CAD files^22^ and in the SI) and new software described in the SI and deposited on GitHub,^24^ which is immediately available as open-source material, we hope to facilitate uptake of ultra-high-throughput screening in droplets across the community and make it the method of choice for protein engineering by directed evolution.

## Supporting Information Available

Video of a sorting experiment (AVI)
Detailed protocols, droplet sorting algorithm, sorting parameters, postprocessing algorithms, calibration curve, absorption spectrum, droplet histograms, functional assay means, summary of improvements compared to previous design (PDF)
CAD file of the device used (ZIP)

## Supplementary Material

ac2c04144_si_001.avi
