# Supplementary material for: Ultra-High-Throughput Absorbance-Activated Droplet Sorting for Enzyme Screening at Kilohertz Frequencies
Source: Anal Chem. 2023 Feb 27;95(10):4597–604. doi: 10.1021/acs.analchem.2c04144 (PMC10018449; doi:10.1021/acs.analchem.2c04144)
Supplement: Supplementary file 2 — ac2c04144_si_002.pdf [file ac2c04144_si_002.pdf]

Supporting Information

Ultrahigh-throughput Absorbance Activated Droplet Sorting (UHT-AADS) for enzyme screening at kilohertz frequencies

Elliot J. Medcalf[1], Maximilian Gantz[1], Tomasz S. Kaminski[1,2,*], Florian Hollfelder[1,*]

1 - Department of Biochemistry, University of Cambridge 80 Tennis Court Road, CB2 1GA Cambridge (UK)

2- Department of Molecular Biology, Institute of Biochemistry, Faculty of Biology, University of Warsaw, Miecznikowa 1, 02-096 Warsaw (Poland)

*Correspondence to fh111@cam.ac.uk, ts.kaminski2@uw.edu.pl

Table of Contents

1. Detailed microfabrication protocol.
2. Parameters for sorting.
3. Detailed protocol for the enrichment experiment.
4. Detailed improvements in the sorting algorithm and electronics.
5. Droplet sorting algorithm.
6. Droplet sorting algorithm throughput maximum.
7. Droplet detection post-processing script and graph generator (no edges).
8. Droplet detection post-processing script and graph generator (edges).
9. Sensitivity of detection.
10. Absorption spectrum of 1,3-bis(trifluoromethyl)-5-bromobenzene.
11. Absorbance values at 455 nm of droplets for various concentrations of 1,3-bis(trifluoromethyl)-5-bromobenzene.
12. Histograms of selection output.
13. Mean absorbance at 340 nm for enrichment experiment.
14. Summary of the improvements of the UHT-AADS compared to the previous designs.
15. References.

1. Detailed microfabrication protocol

1.1 Photolithography of microfluidic moulds

The channel layout for the microfluidic chips was designed using AutoCAD (Autodesk) and printed out on a high-resolution film photomask (Micro Lithography Services). The designs in Fig. 1 are deposited on https://openwetware.org/wiki/Dropbase:_UHT-AADS_Sorter. The microfluidic devices were fabricated following standard hard lithography protocols that can be performed in the local cleanrooms or outsourced to contract manufacturing companies. First, microfluidic moulds were patterned on 3" size silicon wafers (Microchemicals) using high-resolution film masks (Microlithography Services Ltd) and SU-8 2050 photoresists (Kayaku Advanced Materials). A MJB4 mask aligner (SÜSS MicroTec) was used to UV expose all the SU-8 spin-coated wafers. The thickness of the structures (corresponding to the depth of channels in the final microfluidic devices) was measured using a DektakXT Stylus profilometer (Bruker).

Table S1 Fabrication steps with detailed instructions.

	**Fabrication step**	
	**1st layer**	**2nd layer**
Nominal thickness	50 µm	50 µm 2nd layer (100 µm final thickness)
Resist used	SU-8 2050	SU-8 2050
Spin coating speed	1st step: 10 sec, 500 rpm<br>2nd step: 30 sec, 3500 rpm	1st step: 10 sec, 500 rpm<br>2nd step: 30 sec, 4300 rpm
Pre-baking	3 min at 65°C<br>6 min at 95°C	3 min at 65°C<br>6 min at 95°C
Exposure (at ~10 mW cm^{2})	2 x 8.5 sec	2 x 8.5 sec
Post-baking	1 min at 65°C<br>6 min at 95°C	1 min at 65°C<br>6 min at 95°C
Development in the beaker filled with 30-50mL of PGMEA (propylene glycol methyl ether acetate, Sigma-Aldrich)	Approximately 3-5 minutes until all uncured SU-8 is removed from the wafer; development time depends on the intensity of manual agitation. The development step after the 1st deposition is performed only for a 1-layer chip.	Approximately 5-10 minutes until all uncured SU-8 is removed from the wafer, development time depends on the intensity of manual agitation.
Hard baking (optional)	5 min at 150 ^{o}C (only for a 1-layer chip)	5 min at 150 ^{o}C
Measured range of thicknesses	46-47 µm	96-97 µm

1.2 Soft lithography

To manufacture PDMS microfluidic devices, 20-30 grams of a silicone elastomer base and a curing agent (Sylgard 184, Dow Corning) were mixed at a 10:1 (w/w) ratio in a plastic cup and degassed in a vacuum chamber for 30 minutes. PDMS was then poured on a SU-8 master wafer with SU-8 structures and cured in the oven at 65ºC for at least 4 hours. Next, the inlet holes were punched using 1.0 mm diameter biopsy punchers with plungers (Kai Medical Industries). The patterned PDMS chip was then plasma bonded first to an approximately 1-mm-thin PDMS slab (cured beforehand) and then to a 52 mm x 76 mm x 1 mm (length x width x thickness) glass slide (VWR) in a low-pressure oxygen plasma generator (Femto, Diener Electronics). As a result, we obtained a 3-layer device with patterned PDMS on top, a thin PDMS slab in the middle, and a glass slide at the bottom. Next, the hydrophobic modification of microfluidic channels was performed by flushing the device with 1% (v/v) trichloro(1H,1H,2H,2H-perfluorooctyl)-silane (Sigma-Aldrich) in HFE-7500 (3M) and baked on a hot plate at 75 °C for at least 30 minutes to evaporate the fluorocarbon oil and silane mix.

1.3 Integration of optical fibres with the sorting chip

The fabrication of the sorting chip required additional integration with incident light and detection multimode optical fibres with SMA connector type, cladding diameter of 125 µm and a core diameter of 50 µm with a numerical aperture (NA) of 0.22. A single 5m-long fibre optic patch cable (cat. no M14L05, Thorlabs) was cut at the middle, and the outer protective PVC jacket was removed using a three-hole fibre stripper (cat. no FTS4, Thorlabs). Next, the Kevlar protective threads were cut with a scalpel and finally, acrylate coating was removed using a fibre stripping tool (cat. no T06S13, Thorlabs). In the next step, the tip of the fibre tip was cleaved using a ceramic fibre scribe (cat. no CSW12-5, Thorlabs) in order to obtain a flat tip end. The quality of the cleavage was inspected by passing a low power light through the fibre and a visual inspection of the shape of a beam emerging from the fibre tip end. If necessary, the cleavage was repeated until the spherical shape of the light beam was observed. The fibre ends were fixed to the microfluidic chip at least 1 day prior to an experiment. The fibre fixing process was performed on the microscope stage, and a microscope camera was used to verify the position of the fibre ends. First, the microfluidic channels housing the fibres were filled with liquid PDMS and then fibre tips were manually inserted into the chip. Fibres were additionally stabilized by attaching them to the glass slide of the chip with epoxy glue (Araldite® Two Component Epoxy Paste Adhesive). The whole chip was left overnight on the microscope stage to let the PDMS and glue cure at room temperature. Such chips can be used for several sorting experiments, provided that they are washed carefully with pure HFE-7500 and dried by blowing compressed air over them after each use.

2. Parameters for sorting

Table S2 Flow rate for droplet sorting.

Frequency [Hz]	Droplets [µl/min]	RI-matching oil [µl/min]	Bias oil (side channel) [µl/min]
1000	5.75	57.50	28.75
1500	8.00	88.00	44.00
2000	11.50	115.00	57.50

Table S3 Electronic parameters for droplet sorting.

Frequency [Hz]	Period [ms]	Pulse width [µs]	Pulse delay [µs]	Voltage [kV]
1000	10	~1500	~3500	1
1500	10	~1250	~1750	1
2000	10	~750	~1250	1

3. Detailed protocol for the enrichment experiment

3.1 Enrichment of a Variant Expressing Phenylalanine Dehydrogenase

BL21(DE3) competent *E. coli* (New England Biolabs (NEB)) was transformed with a pASK-IBA63plus vector (IBA Lifesciences) harbouring a wild type PheDH strep tag fusion construct (Gielen *et al.*[1]) as a positive control, and a glycosidese as a negative control. The cells were grown to an OD_{600} of 0.4 – 0.6 in LB media, and expression was induced by adding 200 ng/µl anhydrotetracycline. The proteins were expressed for 18-24h at 20°C and 200 rpm. A 1:100 dilution of positive to negative control in 20% Percoll (SigmaAldrich) was compartmentalized with substrate solution (4% cell lytic B (SigmaAldrich), 2 µl/ml r-lysozyme (Merck), 20 mM NAD (SigmaAldrich), 20 mM L-Phenylalanine, 15 mM WST-1 (NBS biologicals), 5 µg/ml mPMS and 2 mM tartrazine in 100 mM Glycine KOH buffer pH 8) in a flow focusing droplet generation device[1] at a 1:1 ratio. Droplets were incubated overnight, and the positive fraction was sorted at 1 kHz. After sorting, 1H,1H,2H,2H-perfluorooctanol (Alfa Aesar) was added (1:1 ratio to oil), and the emulsion was broken by vortexing (1 min, full-speed). The oil phase was washed three times with 100 µl 2 ng/µl salmon sperm DNA (Invitrogen). The aqueous phase was purified and transformed into *E. coli* E. cloni 10G cells (Lucigen) by electroporation (1.8 kV). Cells were plated on LB agar supplemented with 100 µg/ml ampicillin and incubated overnight at 37 °C.

3.2 Plasmid recovery and secondary assay

Plasmid DNA was extracted from the cells and transformed into *E. coli* Bl21(DE3) (NEB). Single colonies were picked and grown to saturation in 96 well plates. The cultures were diluted, grown to an OD_{600} of approximately 0.6 and protein expression was induced by the addition of 0.2 mM IPTG. After expression for 18h (room temperature, shaking at 650 rpm), cells were pelleted (centrifugation at 4000 x g for 20 min) and resuspended and incubated in 200 µL lysis buffer (100 mM Glycine KOH buffer pH10, 4 mg/ml egg white lysozyme (Sigma) and 0.5 mg/mL polymyxin B (Sigma)) for 1h (room temperature, 650 rpm). The lysate was cleared by centrifugation. Enzyme assays were conducted in 96-well microplates with 10 mM L-Phenylalanine, 10 mM NAD in 100 mM Glycine KOH pH 10. Absorption at 340 nm was measured after incubation for 20 h.

3.3 Calculation of enrichment factors

For calculating enrichment factors equations previously used by Baret *et al.*[2] and Zinchenko *et al.*[3] were employed.

Baret *et al.* define enrichment ɲ as shown in formula a:

$$\text{(a) } \eta = \frac{N_+^1}{N_-^1} \Big/ \frac{N_+^0}{N_-^0}$$

N_+^1 represents positive clones after sorting, N_-^1 negative clones after sorting, N_+^0 positive clones before sorting and N_-^0 negative clones before sorting.

Zinchenko et al. define enrichment slightly differently (formula b):

$$\text{(b) } \eta' = \frac{N_+^1}{N_+^1 + N_-^1} \Big/ \frac{N_+^0}{N_-^0 + N_+^0}$$

Table S4: Calculation of enrichment values.

N_+^0	N_-^0	N_+^1	N_-^1	ɲ[a]	ɲ’[b]
1	99	34	2	1683	94

4. Detailed improvements in the sorting algorithm and electronics

4.1 Improvement in sorting algorithm

As throughput and speed are improved through the device design, the speed of computation for sorting decisions is challenged. It is important to consider the algorithms for droplet sorting since the maximum speed of sorting cannot be higher than the time needed for computational decision making (determined by the maximal computation speed). Previously the algorithm used in Gielen *et al.*[1] was limited to triggering by a simple threshold value which made setting a stable gate more difficult. The use of a delay function meant that sorting would be limited in speed if a gating step was added. The open-source Arduino software developed in Gielen *et al.*[1] was therefore redesigned (incorporating gating code modifications from Zurek *et.al.*[4]) to include gating of droplet residence times and voltages and the possibility to sort for 'negative' droplets (i.e. droplets with a lower absorbance than the rest of the population) was added. These improvements allow much more precise control over which droplet events are sorted, such that anomalous events (e.g. passing of an air bubble which usually triggered the electrodes due to a decrease in voltage), are identified and deselected due to gating. Likewise merged droplets and other artefactual events (e.g. dust passing the optic fibres) can also be excluded. Additionally, better control over sorting thresholds increases applicability of the setup to directed evolution campaigns where sorting gates need to be strictly controlled to avoid sorting of inactive variants.

To optimize the speed of the code, static variables were converted to a constant integer type, the micros function was used to allow delays without blocking the program, and blocks of code were wrapped in functions. The code has also been designed to run at speeds in line with the increase in droplet throughput. In simulated tests (shown in supplementary 5) of purely electronic response rates, the maximum throughput of the new algorithm is 4 kHz using an Arduino Due device. Custom Python scripts were created to process the raw data from the photodetector. The SciPy package, alongside others, allowed peak detection of droplets, determined the baseline value, and further explored the data.

4.2 Electronics and Arduino Due

The sorting electronics originally designed by Gielen *et al.*[1] included an Arduino Due, which sends a pulse at 5V to a pulse generator which then triggers a Function Generator linked to a Voltage Amplifier to pulse at 10 kHz at 10 V, which is amplified to 1 kV. This avoided the use of an FPGA (Field Programmable Gate Array) to measure input data and trigger pulses. Even though FPGAs enable parallel processing, and their hardware is programmable, together resulting in computation speeds that are higher than microcontrollers, expertise in low-level programming such as Verilog or VHDL is required, limiting both the accessibility and reconfigurability of these devices for the microfluidics community[5]. By contrast, our Arduino-type microcontroller can be programmed using higher-level languages (decreasing complexity and increasing accessibility), are open-source with significant community support, incur low costs and their code can be modified more easily compared to FPGAs. Given that processing complexities for droplet sorting are minimal, and with the increasing processing speed of

microcontrollers, we argue that using an FPGA for most droplet sorting applications is redundant since the extra complexity is not needed.

5. Droplet sorting algorithm

The droplet detection algorithm for Arduino-like microcontrollers including gating for residence time and voltage at high frequencies. This allows for sorting using droplet size and the ability to exclude electrode activation for events such as air bubbles and dust. Additionally, 'negative' sorting can be achieved (selecting for a decrease in absorbance compared to the negative population) through selecting for a minimum voltage. The maximum sorting capabilities for an Arduino Due is around 4 kHz. The code is modified from Zurek *et al.*[4] and is written in the C programming language. The code was written to maximize the speed of computation and run on an Arduino Due. Constant integer types were used, blocks of code were wrapped in functions, and the 'micros' function was used to allow continuous running of code with delays. A PicoScope 3000 Series (Pico Technology) oscilloscope was used to create an arbitrary waveform that resembled droplets passing the optical fibre. To determine the maximum frequency, the output of the Arduino Due was measured using the oscilloscope and the frequency of the arbitrary waveform was increased until the output of the Arduino Due did not trigger at the threshold value. New software is deposited on GitHub (https://github.com/fhlab/UHT-AADS).

```
//Enter Values Here
const float sortThresh = 6; // in volts
const float dropletThresh = 2; // in volts
const float minVoltage = 4; // in voltz
const int minResidence = 2; // in microseconds
const int maxResidence=5000; // in microseconds
const int outputSource = 13; //connect this pin to function generator
const int inputSource = A0; //connect photodetector input to this pin

//Other Global Variables
float absRead;
long dropletWidth;
long dropletTimerEnd;
long dropletTimerStart;

//absorbance read
float reading(){
absRead = analogRead(inputSource);
absRead = absRead/4096*10; //change 4096 depending on analogReadResolution
return absRead;
}
```

```
void setup() {
  Serial.begin(9600);
  pinMode(outputSource, OUTPUT);
  analogReadResolution(12);
 REG_ADC_MR = (REG_ADC_MR & 0xFFF0FFFF) | 0x00020000; //faster analog
read
}

// Pulses according to parameters
void pulse(){
delayMicroseconds(50);
digitalWrite(outputSource, HIGH);
delayMicroseconds(50);
digitalWrite(outputSource, LOW);
}

// Advanced sorting algorithm
void sortAdv(){
  absRead = reading();
  if (absRead < dropletThresh){
    if (absRead < sortThresh){
      while (absRead < dropletThresh && absRead > minVoltage){
        absRead = reading();
      }
      dropletTimerEnd = micros();
      dropletWidth = dropletTimerEnd-dropletTimerStart;
      if (dropletWidth > minResidence && dropletWidth < maxResidence){
          pulse();
      }
    }
  }
  else {
    dropletTimerStart = micros();
  }
}

// Main loop
void loop(){
  sortAdv();
}
```

6. Droplet sorting algorithm – maximum throughput

The droplet sorting algorithm was tested by simulating droplet events and seeing if it could run through the program and output a signal at different frequencies. A Picoscope 3203D (Pico technologies) was used to generate a trigger signal and measure the voltage from the Arduino Due output and the trigger. As can be seen in the Fig S1. the algorithm is capable of outputting a signal up to 4 kHz. At 4.5 kHz the resultant output signal is erroneous, shown by the gaps in the signal. The period was measured using the PicoScope software for the Arduino output at the different frequencies and is shown in the table S5

1 kHz

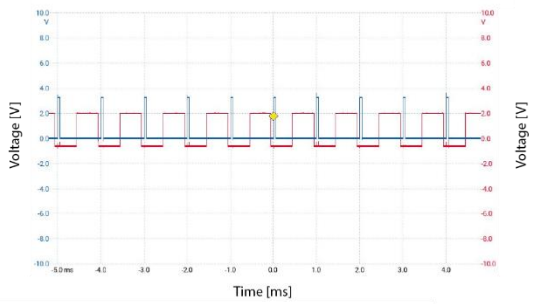

2 kHz

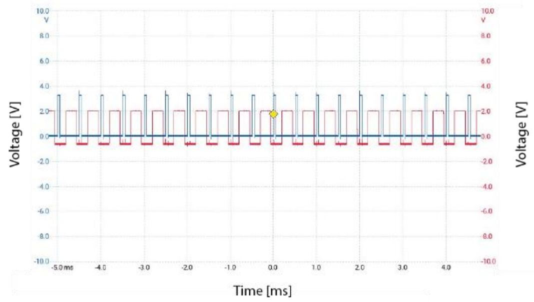

3 kHz

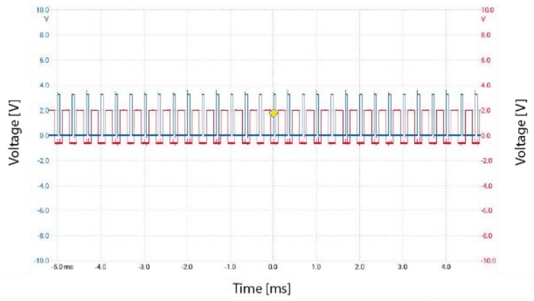

4 kHz

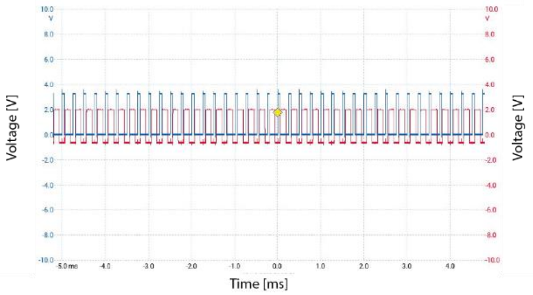

4.5 kHz

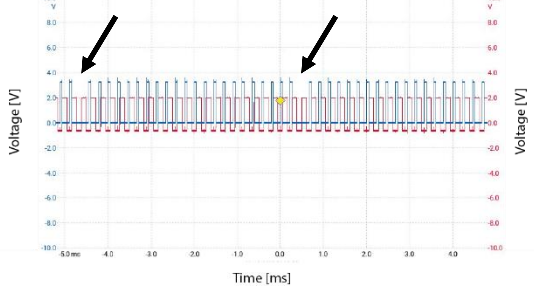

Fig. S1. Plots showing voltage (V) and time (ms) for different throughputs of artificially generated signals. Red is the voltage generated to trigger the Arduino and blue is the voltage corresponding to the Arduino output. As can be seen, the algorithm is capable of running up to 4 kHz on an Arduino Due. However, it cannot respond quickly enough at 4.5 kHz shown by the gaps in the output signal (shown by the arrows).

Table S5: Period measurements from Arduino Due output.

**Frequency [Hz]**	**Expected period [μs]**	**Measured period [μs]**
1000	1000	1000
2000	500	500
3000	333.33	333.33
4000	250	250
4500	222.22	208

7. Droplet detection post-processing script and graph generator (no edges)

The post-processing script for detection of droplet peaks and extraction of data including: number of droplets, frequency, baseline value, number of droplets above the threshold, standard deviation of droplets above the threshold, and gating data. Additionally graphs are produced with and without gating showing the raw data, a histogram plot of droplets and residence time against voltage. The SciPy package[6] was used to infer droplet data and is therefore compatible with future updates to this package. The code was written in the Python programming language.

```
import pandas as pd
import peakutils.baseline
from matplotlib import pyplot as plt
import matplotlib.gridspec as gridspec
import scipy.signal
import numpy as np


droplet_threshold = 9.9 #adjust to desired threshold
hits_threshold = 9.7 #adjust to desired threshold
distance_between_peaks = 20 # number of samples between neighbouring peaks
relative_height = 0.5 # the relative height at which peak width is measured, e.g. 0.5 is
at half the prominence height
sample_rate = 263.000000/9994  #sample rate of detector
bins = 100 # number of histogram bins
residence_time_low = 0.1 #minimum residence time for gating in microseconds
residence_time_high = 0.3 #maximum residence time for gating in microseconds

file = "INSERT FILE" # file should be a csv with a “Time” column and a “Voltage”
column with headers
df = pd.read_csv(file)
df = df[5000:6000] # select data range or comment out to use all data

#convert to pandas dataframe
Time = df["Time"]
Time = Time.to_numpy()
total_time = Time[-1]
total_time_s = total_time / 1000
print("Time interval measured:",np.round(total_time,decimals = 3), "milliseconds")
Voltage = df["Voltage"]
Voltage = Voltage.to_numpy()

#baseline detection
baseline = peakutils.baseline(-Voltage)
average_baseline = -np.mean(baseline)
```

```
print("Baseline at:", np.round(average_baseline, decimals = 3))

#peak detection
minima_V = Voltage*-1
minima =
scipy.signal.find_peaks(minima_V,distance=distance_between_peaks,height=-
droplet_threshold)
minima_sort =
scipy.signal.find_peaks(minima_V,distance=distance_between_peaks,height=(-
hits_threshold,-9.25))
points, _ = minima
points_sort, _ = minima_sort
min_pos = Time[minima[0]]
min_height = minima_V[minima[0]]
peak_widths = scipy.signal.peak_widths(minima_V,points,
rel_height=relative_height)
widths = peak_widths[0]
residence_time = widths*sample_rate

#graphs without gating

gs = gridspec.GridSpec(2,2, wspace = 0.5, hspace = 0.5, top = 0.94)

#voltage against time
fig = plt.figure()
ax1 = fig.add_subplot(gs[1,:])
ax1.plot(Time,Voltage)
ax1.scatter(min_pos,min_height*-1,color='gold',s=50,marker='X')
plt.xlabel("Time [ms]")
plt.ylabel("Detection Signal [V]")
ax1.grid()

#voltage against residence time
ax2 = fig.add_subplot(gs[0,1])
plt.scatter(residence_time,Voltage[points], alpha=0.1)
plt.xlabel("Residence time [us]")
plt.ylabel("Voltage [V]")

#histogram of droplet detection signals [V]
ax3 = fig.add_subplot(gs[0,0])
plt.hist(min_height*-1,bins=bins)
plt.xlabel("Detection signal [V]")
plt.ylabel("Relative frequency")
fig.suptitle(file, fontsize = 8)
plt.show()

#printing of detected data
frequency = points.size/total_time_s
```

```
print(np.around(frequency,decimals = 1),"droplets per second")
print(points.size, "droplets detected")
print(points_sort.size,"droplets detected above threshold")
V_values_above_threshold = Voltage[points_sort[:99]]
print("Standard deviation of droplet voltages detected above
threshold:",np.round(np.std(V_values_above_threshold), decimals = 3), "volts")

#peak detection for gating and printing of detected data
peak_widths = scipy.signal.peak_widths(minima_V,points_sort,
rel_height=relative_height)
widths = peak_widths[0]
residence_time = widths*sample_rate
residence_time_gated = residence_time > residence_time_low
residence_time_gated = residence_time[np.where((residence_time >=
residence_time_low) & (residence_time <= residence_time_high))]
points1 = points_sort[np.where((residence_time > residence_time_low) &
(residence_time < residence_time_high))]
gated_voltage = Voltage[points1]
print(len(residence_time_gated),"droplets detected with gate")

#graphs with gating

#gated histogram of droplet detection signals
gs = gridspec.GridSpec(1,2, wspace = 0.5, hspace = 0.5, top = 0.94)
fig = plt.figure()
ax4 = fig.add_subplot(gs[:,1])
plt.hist(gated_voltage,bins=bins)
plt.xlabel("Detection signal [V]")
plt.ylabel("Relative frequency")
plt.title("Voltage with Gated Residence Time")

#gated voltage against residence time
ax4 = fig.add_subplot(gs[0,0])
plt.scatter(residence_time_gated,gated_voltage, alpha=0.5)
plt.xlabel("Residence time [us]")
plt.ylabel("Voltage [V]")
plt.title("Gated Residence Time")
plt.show()
```

8. Droplet detection post-processing script and graph generator (edges)

The post-processing script for detection of droplet peaks and extraction of data as above, but for when peaks are masked by droplet edges.

```
import numpy as np
from scipy.signal import argrelextrema
import scipy.signal
import pandas as pd
import peakutils.baseline
from matplotlib import pyplot as plt

file = "Insert file"
df = pd.read_csv(file)
df = df[100000:101000]

Time = df["Time"]
Time = Time.to_numpy()
total_time = Time[-1]
total_time_s = total_time / 1000
print("Time interval measured:",np.round(total_time,decimals = 3), "milliseconds")
Voltage = df["Voltage"]
Voltage = Voltage.to_numpy()

baseline = peakutils.baseline(-Voltage)
average_baseline = -np.mean(baseline)
print("Baseline at:", np.round(average_baseline, decimals = 3))

threshold_max_higher_lower_bounds = 10.2
# for local maxima
local_max_higher = argrelextrema(Voltage, np.greater)[0]
local_max_higher =
local_max_higher[(Voltage[local_max_higher]>threshold_max_higher_lower_bounds
)]
threshold_max_lower_upper_bound = 7
threshold_max_lower_lower_bounds = 2
# for local maxima
local_max_lower = argrelextrema(Voltage, np.greater)[0]
local_max_lower =
local_max_lower[(Voltage[local_max_lower]>threshold_max_lower_lower_bounds)]
local_max_lower =
local_max_lower[(Voltage[local_max_lower]<threshold_max_lower_upper_bound)]
# for local minima
local_min = argrelextrema(Voltage, np.less)[0]
local_min = local_min[(Voltage[local_min]<8)&(Voltage[local_min]>4)] #change
numerical values to match dataset
```

```
local_max = np.append(local_max_higher,local_max_lower)

fig = plt.figure(figsize=(8,4))

plt.plot(Time,Voltage)
plt.scatter(Time[local_min],Voltage[local_min],color='gold',s=50,marker='X')
plt.scatter(Time[local_max],Voltage[local_max],color='gold',s=50,marker='X')
fig.suptitle(file, fontsize = 8)
plt.autoscale(enable=True, axis='x', tight=True)
plt.show()
```

9. Sensitivity of detection

Recalibration of the sensitivity is needed in reference to Gielen *et al.*[1] since the absorbance values are affected by the smaller path length. The range of the calibration plot was generated by converting to the mean absorbance for 2000 droplets (Fig. S2). As can be seen, there is a positive linear trend as the concentration of tartrazine increases from 100 µM to 5 mM, as expected. Below this, the trend does not continue showing that the device's sensitivity is around 100 µM. Since smaller droplets are used to sort at higher frequencies, the path length is decreased, and in accordance with the Beer-Lambert law, the dynamic range is decreased. The advantages, however with smaller droplets, as previously mentioned, are a higher throughput, a higher local concentration of potential enzyme, and a smaller amount of chemical solutions needed. As can be seen in Fig. S1, the standard deviation of the absorbance values is small, indicating that the precision of detection is high.

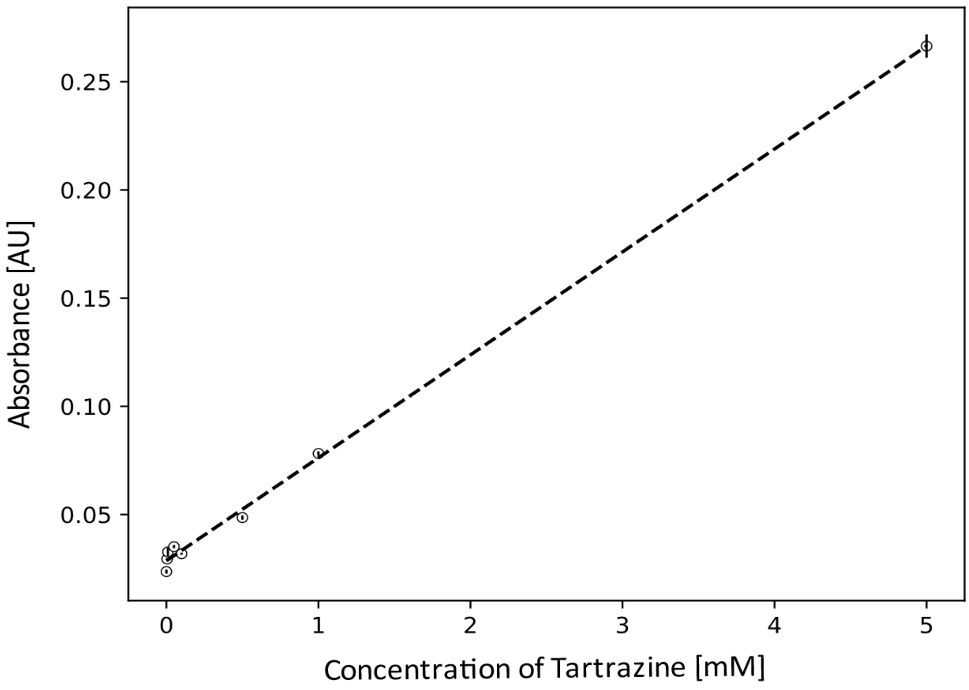

Fig. S2. Calibration plot showing the linear relationship of absorbance to the concentration of tartrazine (mM) which is used as a model absorbance compound. A residence time gate was applied to remove artefacts such as air bubbles or merged droplets. Two milliseconds of data (around 2000 droplets with a volume of 75 pL) were measured, and the peak values were determined. Absorbance was calculated using the equation $A = -log10(V_n/V_0)$, where V_n is the voltage at a given concentration and V_0 is the voltage for pure RI-matching oil. 35% RI-matching oil was used for spacing of droplets. Error bars are the standard deviation of the ~2000 droplets.

10. Absorption Spectrum of 1,3-bis(trifluoromethyl)-5-bromobenzene

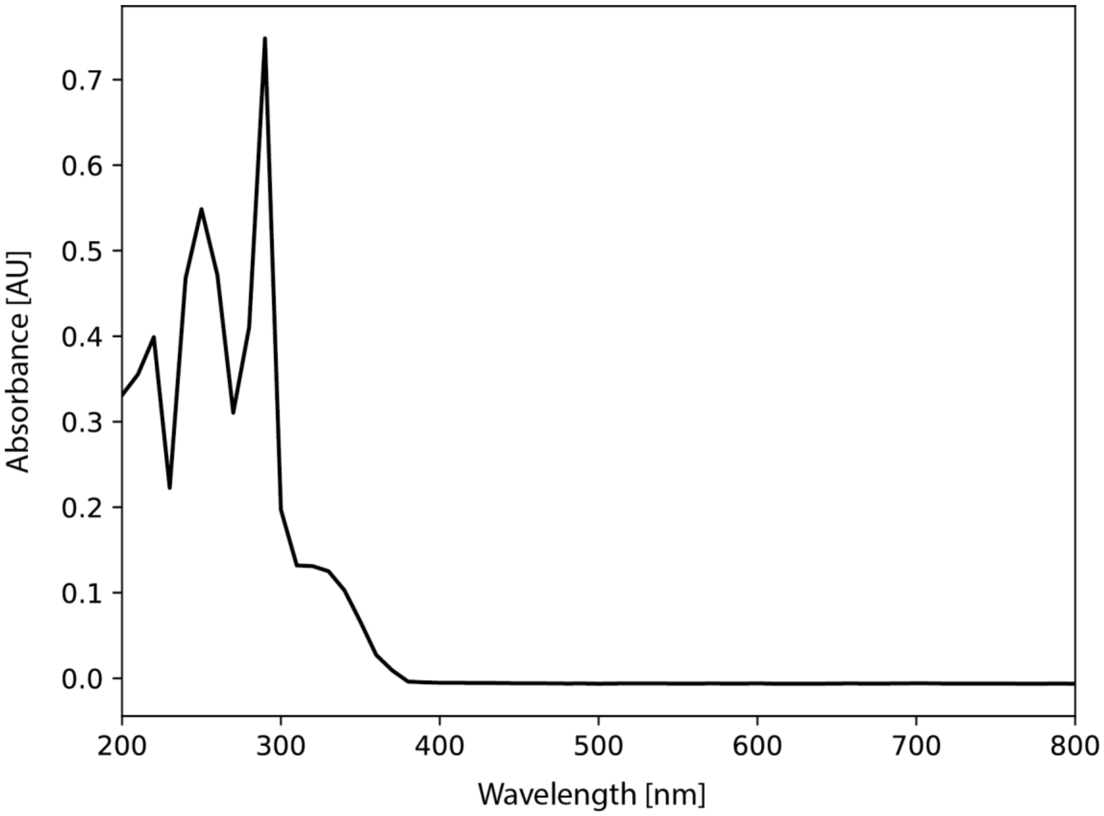

Fig. S3. The absorbance spectrum of 1,3-bis(trifluoromethyl)-5-bromobenzene. The compound absorbs in the UV range but does not in the visible range.

11. Absorbance values at 455 nm of droplets for various concentrations of 1,3-bis(trifluoromethyl)-5-bromobenzene

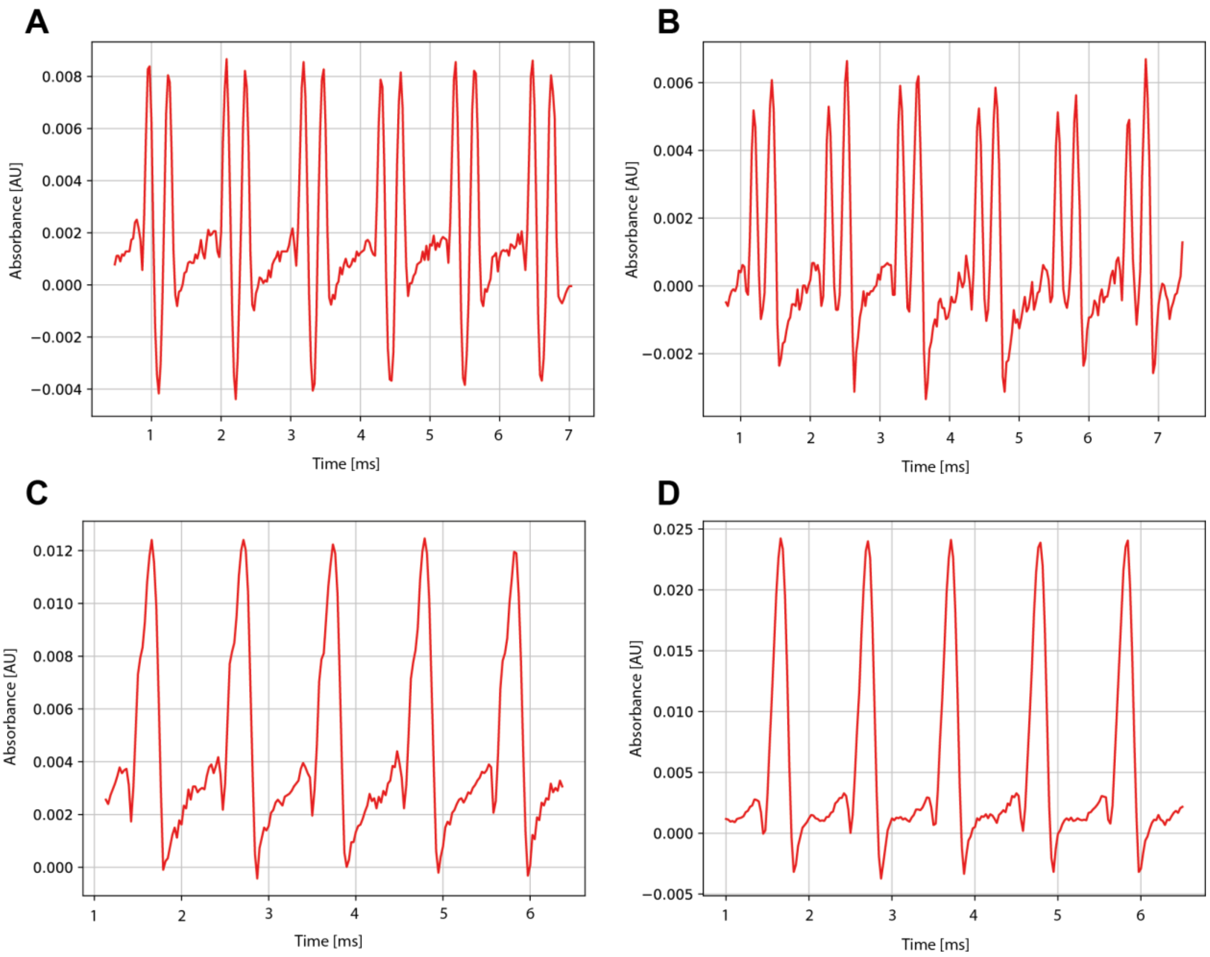

Fig. S4. The absorbance traces for droplets (water) using various percentages of 1,3-bis(trifluoromethyl)-5-bromobenzene mized with HFE-7500. Percentages are the percentage volume of 1,3-bis(trifluoromethyl)-5-bromobenzene added to HFE-7500 oil. **(A)** 25%, **(B)** 27.5%, **(C)** 30%, **(D)** 35%. 35% produces distinguishable peaks without any edges.

12. Histograms of selection output

The distribution function of data for 75 pL droplets for the experiment shown in Fig. 3. showing the separation between the two different droplet populations.

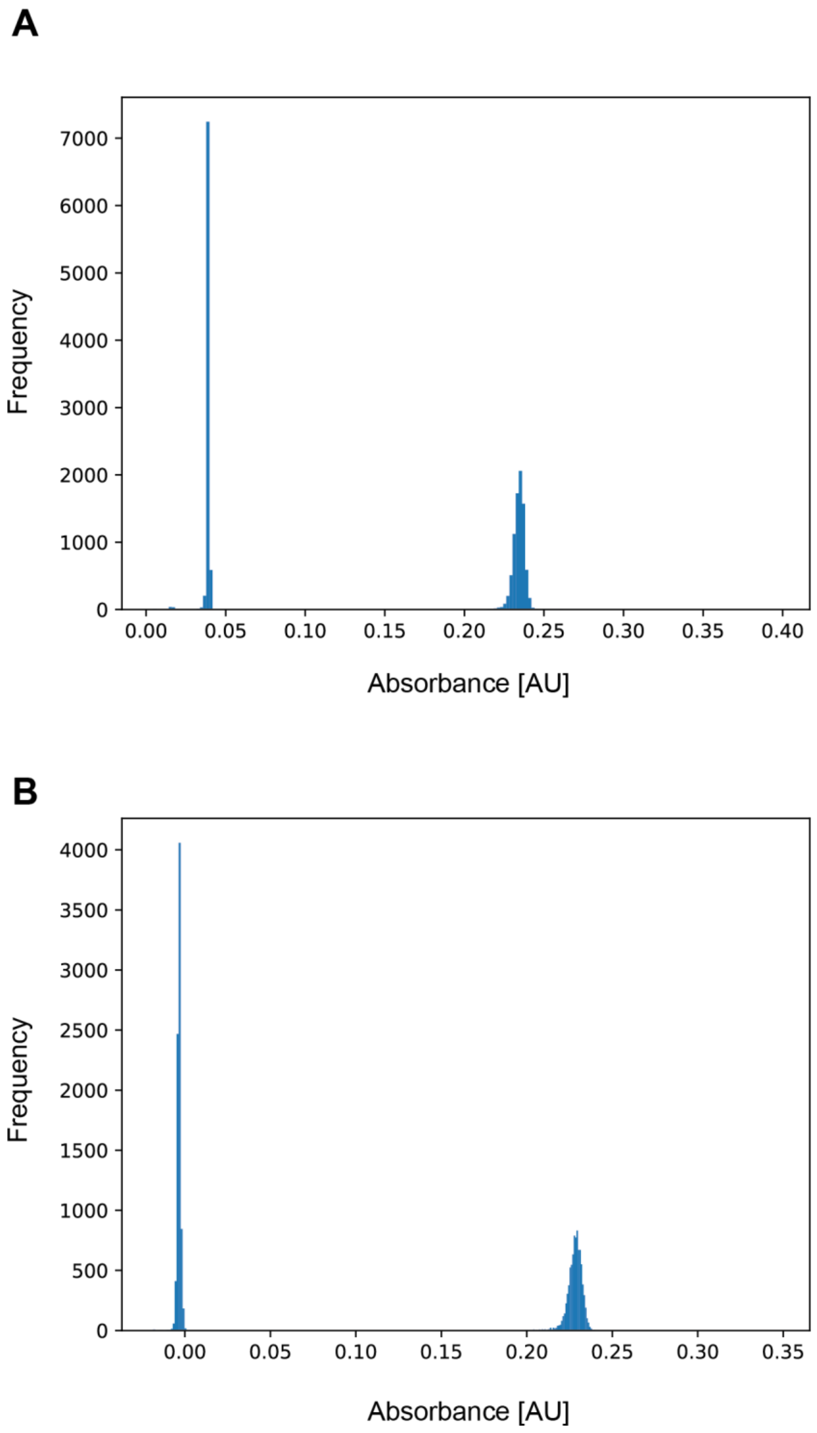

Fig. S5. Histograms showing the absorbance at 455 nm for two droplet populations, 0.5 mM tartrazine, and 5 mM tartrazine. **(A)** With RI-matching compound at 35%. 0.5 mM mean = 0.41, standard deviation = 0.0064, 5 mM mean = 0.24, standard deviation = 0.0015. **(B)** Negative control (HFE-7500 with 2% RAN surfactant). 0.5 mM mean = -0.001, standard deviation = 0.014, 5 mM mean = 0.23, standard deviation = 0.011. 15 seconds of data with a droplet freuquency of 1000 Hz were used.

13. Absorbance values for functional screen

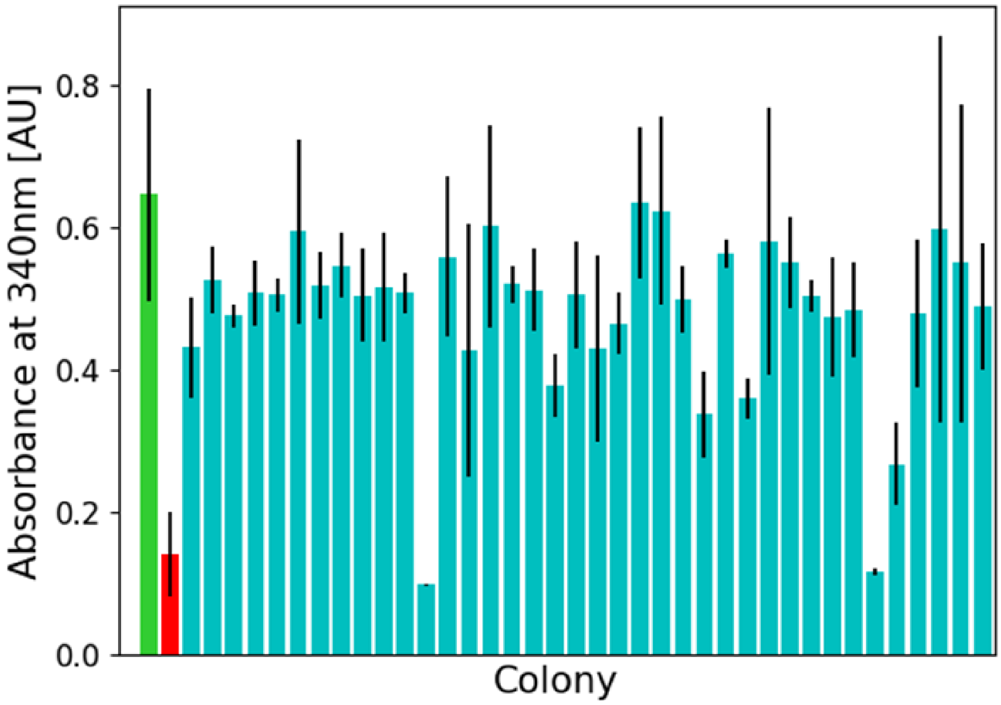

Fig. S6. Mean absorbance at 340 nm for 38 colonies (cyan, n=3 for each) picked after transformation of DNA collected from the positive outlet of the AADS device sorting at one kilohertz. The green bar shows the mean (n=9) of the positive control (pASK expressing wild type PheDH) and the red bar shows the mean (n=9) of the negative control (pASK expressing glycosidase). Error bars represent the standard deviation.

14. Summary table of improvements

Table S6. Summary of the improvements of the UHT-AADS compared to the previous designs (Gielen *et al.* [1], Zurek *et al.* [4]).

**UHT-AADS Addition compared to AADS**	**Improvement**
Faster sorting speed	Combination of points below to achieve sorting at 1000 Hz with 100% efficiency for the 100 videos analyzed, a 10-fold increase of throughput.
RI-matching oil	Removes scattering signal from droplet edges in the recorded traces and allows detection of droplets with a lower concentration of substrate and reduced volume.
Single-layered inlet	Allows separation of smaller droplets individually and to droplets be evenly spaced.
Bias oil	Acts as a barrier to droplets from entering the positive outlet without dielectrophoresis. Allows transfer of positive hits to oil and surfactants solution dedicated for storage of stable emulsion
Faster Sorting algorithm with gating	Sorting algorithm capable of sorting at 4kHz with an Arduino Due. Adds sorting gates to select for specific residence time and voltage ranges.

15. Supplementary References

(1) Gielen, F.; Hours, R.; Emond, S.; Fischlechner, M.; Schell, U.; Hollfelder, F. Ultrahigh-Throughput–Directed Enzyme Evolution by Absorbance-Activated Droplet Sorting (AADS). *Proc National Acad Sci* 2016, *113* (47), E7383–E7389. https://doi.org/10.1073/pnas.1606927113.

(2) Baret, J.-C.; Miller, O. J.; Taly, V.; Ryckelynck, M.; El-Harrak, A.; Frenz, L.; Rick, C.; Samuels, M. L.; Hutchison, J. B.; Agresti, J. J.; Link, D. R.; Weitz, D. A.; Griffiths, A. D. Fluorescence-Activated Droplet Sorting (FADS): Efficient Microfluidic Cell Sorting Based on Enzymatic Activity. *Lab Chip* 2009, *9* (13), 1850. https://doi.org/10.1039/b902504a.

(3) Zinchenko, A.; Devenish, S. R. A.; Kintses, B.; Colin, P.-Y.; Fischlechner, M.; Hollfelder, F. One in a Million: Flow Cytometric Sorting of Single Cell-Lysate Assays in Monodisperse Picolitre Double Emulsion Droplets for Directed Evolution. *Anal Chem* 2014, *86* (5), 2526–2533. https://doi.org/10.1021/ac403585p.

(4) Zurek, P. J.; Hours, R.; Schell, U.; Pushpanath, A.; Hollfelder, F. Growth Amplification in Ultrahigh-Throughput Microdroplet Screening Increases Sensitivity of Clonal Enzyme Assays and Minimizes Phenotypic Variation. *Lab Chip* 2020, *21* (1), 163–173. https://doi.org/10.1039/d0lc00830c.

(5) García, G.; Jara, C.; Pomares, J.; Alabdo, A.; Poggi, L.; Torres, F. A Survey on FPGA-Based Sensor Systems: Towards Intelligent and Reconfigurable Low-Power Sensors for Computer Vision, Control and Signal Processing. *Sensors* 2014, *14* (4), 6247–6278. https://doi.org/10.3390/s140406247.

(6) Virtanen, P.; Gommers, R.; Oliphant, T. E.; Haberland, M.; Reddy, T.; Cournapeau, D.; Burovski, E.; Peterson, P.; Weckesser, W.; Bright, J.; Walt, S. J. van der; Brett, M.; Wilson, J.; Millman, K. J.; Mayorov, N.; Nelson, A. R. J.; Jones, E.; Kern, R.; Larson, E.; Carey, C. J.; Polat, İ.; Feng, Y.; Moore, E. W.; VanderPlas, J.; Laxalde, D.; Perktold, J.; Cimrman, R.; Henriksen, I.; Quintero, E. A.; Harris, C. R.; Archibald, A. M.; Ribeiro, A. H.; Pedregosa, F.; Mulbregt, P. van; Contributors, S. 1 0; Vijaykumar, A.; Bardelli, A. P.; Rothberg, A.; Hilboll, A.; Kloeckner, A.; Scopatz, A.; Lee, A.; Rokem, A.; Woods, C. N.; Fulton, C.; Masson, C.; Häggström, C.; Fitzgerald, C.; Nicholson, D. A.; Hagen, D. R.; Pasechnik, D. V.; Olivetti, E.; Martin, E.; Wieser, E.; Silva, F.; Lenders, F.; Wilhelm, F.; Young, G.; Price, G. A.; Ingold, G.-L.; Allen, G. E.; Lee, G. R.; Audren, H.; Probst, I.; Dietrich, J. P.; Silterra, J.; Webber, J. T.; Slavič, J.; Nothman, J.; Buchner, J.; Kulick, J.; Schönberger, J. L.; Cardoso, J. V. de M.; Reimer, J.; Harrington, J.; Rodríguez, J. L. C.; Nunez-Iglesias, J.; Kuczynski, J.; Tritz, K.; Thoma, M.; Newville, M.; Kümmerer, M.; Bolingbroke, M.; Tartre, M.; Pak, M.; Smith, N. J.; Nowaczyk, N.;

Shebanov, N.; Pavlyk, O.; Brodtkorb, P. A.; Lee, P.; McGibbon, R. T.; Feldbauer, R.; Lewis, S.; Tygier, S.; Sievert, S.; Vigna, S.; Peterson, S.; More, S.; Pudlik, T.; Oshima, T.; Pingel, T. J.; Robitaille, T. P.; Spura, T.; Jones, T. R.; Cera, T.; Leslie, T.; Zito, T.; Krauss, T.; Upadhyay, U.; Halchenko, Y. O.; Vázquez-Baeza, Y. SciPy 1.0: Fundamental Algorithms for Scientific Computing in Python. *Nat Methods* 2020, *17* (3), 261–272. https://doi.org/10.1038/s41592-019-0686-2.
